# Commentary: Nanoscopy reveals the layered organization of the sarcomeric H-zone and I-band complexes

**DOI:** 10.3389/fcell.2020.00074

**Published:** 2020-02-13

**Authors:** Nicanor González-Morales, Frieder Schöck

**Affiliations:** Department of Biology, McGill University, Montreal, QC, Canada

**Keywords:** indirect flight muscle, Drosophila, superresolution imaging, sarcomere, myofibril, muscle structure

Sarcomeres are the smallest contractile units of muscles. They are arranged end-to-end in repetitive arrays forming myofibrils that are anchored to bones or the exoskeleton (Lemke and Schnorrer, 2017). Because muscles are composed of many identical sarcomeres that coordinately contract, understanding their structure and function is critical for understanding muscle biology.

The development of superresolution microscopy together with particle averaging methods opened the door to reanalyze known cellular compartments but with a dramatic increase in resolution (Sigal et al., 2018). Szikora et al. (2020) use dSTORM superresolution fluorescence imaging together with a particle averaging algorithm to help solve the structure of the sarcomere, using the indirect flight muscle (IFM) of *Drosophila* (Figure 1). The IFM has the most regular known sarcomeres because they contract only minimally, but with a very high frequency of 200 Hz. Their unusually invariant structure makes them ideally suited for superresolution approaches. The authors mapped the position of dozens of epitopes with a precision of roughly 10 nanometres, unraveling crucial details of sarcomere structure.

**Figure 1:**
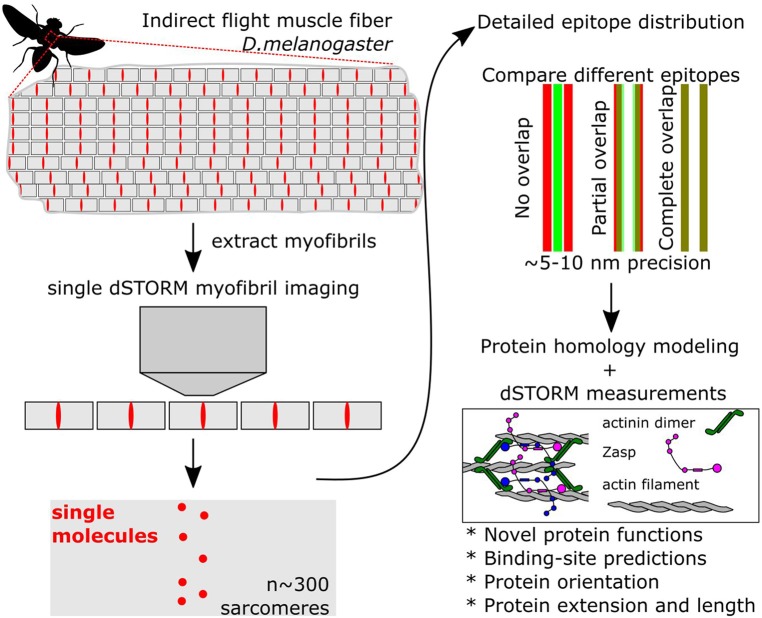
Simplified steps to obtain superresolved images of sarcomere proteins. Muscles are composed of hundreds of myofibrils, composed of repeated sarcomere units. Single myofibrils are extracted from the rest of the muscle. Then, superresolved dSTORM images of single sarcomeres are acquired. Because all sarcomeres are identical, images can be merged using an averaging algorithm. In addition, the localization pattern from different proteins can be directly compared using reference points. By localizing and comparing the position of over 20 proteins Szikora et al. (2020) present a very detailed atlas of the structure of the sarcomere.

Sarcomeres have a region devoid of myosin filaments called I-band and a region devoid of actin filaments called H-zone. In the middle of these regions are big protein complexes that hold the actin or the myosin filaments in place, the Z-disc and the M-line, respectively (Lemke and Schnorrer, 2017).

Szikora et al. (2020) first showed that dSTORM with an averaging algorithm outperformed other superresolution methods. Then, they chose well-characterized and newly made antibodies to map their localization. Initially, the authors mapped the extent of the H-zone and the I-band with antibodies or stains that recognize the myosin heavy chain, filamentous actin, tropomodulin, and actin capping protein. They used these measurements as reference points to map other epitopes. Finally, they made structural homology models of all the tested proteins and matched the predicted models to the localization pattern. Here we highlight four important discoveries and their impact on the general muscle biology field.

## Filamin Orientation at the I-Band

Filamin is a dimeric protein that consists of 22–24 Ig-like domains and two actin-binding domains. Filamin is conserved and mutations in the human filamin-C gene cause severe myopathies. Genetic evidence suggested that in the sarcomere filamin functions as a cohesive bond between actin filaments and titin filaments from opposing sarcomeres that helps resist contractile forces (González-Morales et al., 2017). The new dSTORM images show that the C-terminal region of filamin is located ~50 nm from the center while the N-terminal region lies at the very center of the I-band, providing both the orientation and the length of the filamin dimer. They also explored the localization pattern of Kettin, a homolog of vertebrate titin, using different epitopes, which narrows down the interacting domains of filamin and titin, complementing previous *in vitro* data.

## Lighting Up the Obscurin Protein

The H-zone is understudied compared to the I-band. The M-line, at the middle of the H-zone, is responsible for anchoring myosin filaments. In vertebrates, myomesin and obscurin are the key myosin-binding proteins. In invertebrates obscurin alone fulfills this role. It is a giant titin-like scaffolding protein that localizes to the developing H-zone and mutations in obscurin lead to H-zone disorganization (Katzemich et al., 2012). As obscurin is likely to have a semi-linear structure, the current article explores the localization and orientation of obscurin using two antibodies that recognize the kinase domain near the C-terminal region and the Ig domains 14–16 located in the middle of the protein. They predict obscurin to dimerize at the center of the H-zone through its C-terminal domains and to possibly extend all the way up to the start of the actin filaments that may overlap with its N-terminal region. This would have to be confirmed with an N-terminal obscurin antibody but could indicate a role for obscurin in registering thin filaments, in addition to thick filaments.

## The Very Complex Network of Actin Regulators

Not surprisingly, a very complex network of actin regulators is present in the sarcomere, at both the I-band and H-zone. The current article explores the localization pattern of several actin regulators, including profilin, SALS, and the formins DAAM and Fhos. In contrast to non-muscle cells, actin filaments elongate from their pointed ends facing the H-zone (Mardahl-Dumesnil and Fowler, 2001). The authors provide support for the notion that monomeric actin is present at the H-zone, because they can detect it using fluorescent DNAse-I; profilin then feeds G-actin to formin which may elongate thin filaments by adding short actin polymers at the pointed ends. While actin regulation at the sarcomere is far from solved, the current article gives the exact position of many actin regulators at both the I-band and H-zone and complements previous formin loss-of-function studies (Molnar et al., 2014; Shwartz et al., 2016).

## Actinin and Zasp at the Z-Disc Core

The actinin dimer is the main actin crosslinker at the Z-disc. The authors discovered that actinin forms two distinct bands, suggesting two actinin crosslinking sites at the IFM Z-disc. Then, they tested Zasp52, a known actinin-interacting protein (Jani and Schöck, 2007; Katzemich et al., 2013; Liao et al., 2016). Surprisingly, they found that Zasp52 is located in between the two actinin bands. Because the Zasp52 epitope is located far from the actinin binding site, they propose an orientation of Zasp52 where its N-terminal region is pointing outward, and the C-terminus is at the very center of the Z-disc. This arrangement suggests a crucial role for Zasp52, potentially coordinating the spacing between actinin dimers owing to its own ability to dimerize and oligomerize (González-Morales et al., 2019).

In summary, the work of Szikora et al. (2020) combines the power of superresolution imaging with protein homology modeling to provide an atlas of sarcomere proteins at an unprecedented resolution. Their work will have a huge impact in muscle biology research as it both supports previous functional studies and suggests a plethora of novel testable hypotheses. In that respect analyzing epitope localization by superresolution imaging in many of the mutants already available in the fly or new ones generated by Crispr mutagenesis will be particularly promising.

## Author Contributions

NG-M and FS wrote and edited the manuscript.

### Conflict of Interest

The authors declare that the research was conducted in the absence of any commercial or financial relationships that could be construed as a potential conflict of interest.
